# Supplementary material for: On the problem of boundaries and scaling for urban street networks
Source: J R Soc Interface. 2015 Oct 6;12(111):20150763. doi: 10.1098/rsif.2015.0763 (PMC4614511; doi:10.1098/rsif.2015.0763)
Supplement: Supplementary Information [file rsif20150763supp1.pdf]

Supplementary Information

(Dated: October 9, 2015)

I. THE DATA SET

A. The UK street network

We study the street network in the geographical region defined by Great Britain, excluding Northern Ireland, i.e. the geographical region defined by England, Scotland and Wales which is generally referred to as Britain. The dataset used is *Meridian 2* [1], a publicly available dataset based on street centre lines produced by the UK National Mapping Agency, the Ordnance Survey.

A street network is a planar graph representation of a map, where the street intersections are the vertices N, and the street segments are the links E. In practice, these are extracted from maps given by shape files, where the streets are lines that have been drawn with a mouse. This means that every time there is a "click" a node is generated. Therefore, it is highly likely that a single street will be divided into many different segments with nodes of degree two, in particular for non-straight streets. Nodes of degree two will also appear when a street changes its name. The process of urbanisation can be characterised by the intersection of the streets. We therefore proceed to remove all nodes of degree 2. Note that the main results of the paper would not change if these were left, the extra nodes will only affect the computational process, making it more cumbersome. After that the street network is composed of $N = 715736$ vertices and $E = 1008661$ street segments, with an average degree $\langle k \rangle = 2E/N = 2.82$. For the reasons we explain in Sec.II, we replace all the street segments that represent river bridges with a set of connected segments of 40 m.

Applying for each city the algorithm described in Sec. II A, we extract the 61 largest cities from the dataset. Since the city size distribution approximates a power law, i.e. Zipf's law, it is impossible to set a number of representative cities for this study and for this reason, such a number is arbitrary. In this way, we obtain the UK urban dataset, which is displayed in Fig. S1. All the measures presented in the main text and in the Suppl. Inf. are computed for this specific system. For any plot of urban UK, each point represents an individual green cluster of Fig. S1.

For the analysis, we want to compare two different kinds of networks: rural and urban. In Sec. II A, we have described how to extract a selection of urban street networks. In order to compare these with rural networks, we extract all the possible streets that belong to urbanised areas. We will consider urbanised from as small as 80 intersections. This will ensure that we will not be mixing urban networks in our rural sample. We delete all those cities from the original street network, so that what remains is mostly a rural street network. The so obtained street network is composed of $N = 240274$ vertices and $E = 320425$ edges with an average degree $\langle k \rangle = 2.67$.

The measures on the rural dataset are done by considering 1000 samples of the rural street network, which are extracted by picking a random point in the rural net and by considering the street network contained in a square around this point with a side randomly chosen between 20km and 70km. The large sample size and varying window size for the rural space, together with the different runs ensures the statistical validity of the results.

B. California

We start with the publicly available OSM (Open Street Map) street network for California [2]. We remove from it cycleways, foot-ways and service paths. Finally we keep the largest component, removing the street intersections with degree 2. The resulting network has $N = 1381784$ nodes, and $E = 1850268$ edges, with an average degree $\langle k \rangle = 2.68$.

Applying for each city the algorithm described in Sec. II A, we extract the largest 52 cities of the given dataset. In this way we obtain the urban dataset, whose cities are shown in Fig. S2.

By applying the algorithm of Sec. II A to more clusters, as in the UK case, we extract all the cities with more than 300 intersections. Then, we clear the original dataset from all the extracted cities and in this way we obtain the *rural network* dataset for California, composed of 390473 vertices and 482362 edges for an average degree $\langle k \rangle = 2.47$.

As for the rural UK network, measures for the rural California network are generated by considering 1000 samples of the rural street network, which are obtained by picking up a random point in the rural net and the considering the street network contained in a square around this point with a side randomly chosen between 20km and 70km.

C. The Historical Dataset

For the historical dataset, we employ 9 maps which cover the street network evolution of the Greater London Area from 1786 until 2010, namely 1786, 1830, 1880, 1900, 1920, 1940, 1965, 1990, 2010. The maps were digitalised from original maps with techniques that are described in [3]. We extract from each of these street networks the city cores, with the techniques described in Sec. II A. An analysis of this street network in terms of its primal and dual representation can be found in [3, 4].

It is important to notice that the historical dataset and the urban UK dataset come from different original maps and extraction procedures. The dataset have different

level of detail, and hence one will have more roads than the other, leading to a different "city size" determined by the number of intersection points. Then it is not surprising that the size of modern London, as for instance in Fig. 6 of the main text, differ from a dataset to the other one.

D. Urban Population and Street Intersection Correlations

In Fig. S3, we show the correlations between residential population and street intersections for London throughout its history. On the vertical axis we display the residential population $pop(N)$ for the wards of London from 1786 to 2010 as a function of the street intersections N, for each ward. The aforementioned quantities are strictly correlated and we find that $pop(N) \propto N^{1.2}$.

II. PERCOLATION AND LOGISTIC FIT

Population and population density are the two main variables that have been used in order to define and simulate urban settlements through various techniques including percolation theory, diffusion limited aggregation, and clustering [5–8]. Recently, it has been shown that the street intersection density space is also a good proxy to assess city boundaries [3, 9].

The general strategy used to apply site percolation to the street network, is to consider a geographical area surrounding a city and implement a similar algorithm to the continuous 'City Clustering Algorithm' defined in [7]. Starting from a given distance threshold τ, street intersections which are at a distance smaller or equal than τ are clustered. We expect that increasing τ, larger clusters form until a giant component appears, which spans the street network. The threshold at which the giant component appears is called the critical threshold τ_c and we might be tempted to define the city boundaries as the boundaries of the giant cluster at the percolation threshold.

To find the value of τ_c, we measure the average cluster size N of the cluster which a randomly picked up intersection belongs to (notice that this is different from the average cluster size), when we exclude the largest cluster. This is defined as $N(\tau) = \frac{\sum_s n_s(\tau)s(\tau)^2}{\sum_s n_s(\tau)s(\tau)}$, where $n_s(\tau)$ is the number of clusters of size $s(\tau)$ and it diverges at $\tau = \tau_c$ [10].

The top panels of Fig. S4, show $N(\tau)$ for the street intersection map of London (left panel), and Manchester (right panel). In the case of London, we see how the percolation threshold $\tau_c \approx 205$m appears to be well defined by the point where a discontinuity appears. Unfortunately, this discontinuity corresponds to the threshold at which the south of London merges with the north of London, i.e. the threshold to overcome the River Thames, when the second largest cluster merges with the largest one. To take into consideration such natural barriers, we artificially add intersections in the middle of the bridges. The resulting percolation process is represented by the red curve in the top left panel of Fig. S4. This time the discontinuity disappears, indicating that the threshold previously found was just an artefact derived from the natural barrier.

In order to see whether this behaviour is particular to London, we examine Manchester, which is a morphologically articulated city if compared to London. We find that several natural barriers, such as rivers or green land, are reflected as *jumps* in $N(\tau)$ (see top right panel of Fig. S4).

However, an infinite component does not emerge in these systems, for reasons which will appear clearer at the end of this section.

In the bottom left panel of Fig. S4, following the same percolation procedure, we show the maximum cluster size $N_{Max}(\tau)$ as a function of the threshold for London. The black dots correspond to the case where the River Thames barrier has not been removed, the red points to the case where the river barrier has been removed, by artificially adding street intersections at 40m distance on the bridge segments of the street network. We can see how the behaviour of the red plot is different only for those scales that are smaller than the River Thames width, while for larger scales both behaviours are equivalent. Then we can say that natural barriers do not modify the functional behaviour of $N_{Max}(\tau)$ at large scales. However in order to obtain clear logistic fits for all the cities in the UK, we add intersections at 40m distance on all the bridge segments.

A. Logistic fit algorithm

The present methodology is a bottom-up one. It is specifically designed to extract city boundaries with a high level of precision, but in order to attain such a precision cities need to be analysed singularly. The opposite happens for top-down approaches [11], where a large number of city boundaries can be extracted more efficiently within a certain degree of approximation.

Often small satellite urban conglomerations form around cities. When the city expands, these small towns tend to get absorbed by the city. However, since we observe cities in all their stages of evolution, there are cases where this absorption process is just taking place. This is the case for instance of the city of Manchester, which we show at the top panels of Fig. S5. In such cases, we see that the $N_{Max}(\tau)$ plot seems to be a superposition of different trends. A first condensation process seems to take place defining the city centre ($\tau = 248m$), but just before the condensation, the growth starts again to incorporate the northern extensions of the city. After that the plot eventually condensates for $\tau = 382m$.

Any classical fitting algorithm can be used to extract

the relevant logistic parameters, but in order to automate the city extraction process, we have created an algorithm to find out the best logistic fit for the measured $N_{Max}(\tau)$, i.e. to define the three parameters τ_0, r and C. The first step is to find the inflection point τ_0. To do that, we build the derivative of $N_{Max}(\tau)$, $\Delta N_{Max}(\tau)/\Delta\tau$. This should follow the Hubbert function behaviour, with a peak in τ_0. In order to measure the growth parameter k, we consider the points of $N_{Max}(\tau)$ for $\tau < \tau_0$. These grow exponentially as $e^{r\tau}$. It is important to always take into consideration natural barriers, since these can bias $N_{Max}(\tau)$, i.e. leading to peaks in $\Delta N_{Max}(\tau)/\Delta\tau$. At last, we find the carrying capacity C, by making a logistic fit of $N_{Max}(\tau)$, where τ_0 and r are fixed parameters given by the method hitherto explained. For the reasons we stated before (natural barriers, bridges, etc.), the parameters for the logistic fit extracted through the algorithm could be incorrect. Then a visual check is always performed, and the parameters are re-computed manually if need be.

Another case that we discuss here is the one of Leeds, Bradford and Brighouse in the UK (bottom panels of Fig. S5), which are cities that are in the process of merging, i.e., the urbanization process is leading the cities to merge into a single urban agglomeration.

A simple logistic fit for $N_{Max}(\tau)$ would not lead to an accurate result, since following the normal procedure for the extraction of the condensation threshold, the three cities of Leeds, Bradford and Brighouse are merged into a single urban agglomeration. In such rare cases, a more careful analysis would be required in order to understand what is going on, i.e. the urban merging process, for instance by following the behaviour of the first, second and third largest cluster separately.

Nevertheless the logistic behaviour is present in all the mentioned cases and the apparent functional discongruities can be easily interpreted as stated above.

III. FURTHER STATISTICAL ANALYSIS

Here we show some relationships which could possibly shed some light on the $L(N)$ behaviour shown in the main text. In Fig. S6, in panels *a, c*, we show the average degree $\langle k(N)\rangle$ and the average street length $\langle l(N)\rangle$ as a function of the number of street intersections N for the UK urban street network. As we can see, they are both compatible with a constant function and this relates to the linear behaviour of $L(N)$, as shown in the main text (we remind the reader that we can write $L(N) = \langle k(N)\rangle\langle l(N)\rangle N$). In panels *b, d*, we show the same quantities as measured for the rural UK street network, and we can observe a more inhomogeneous behaviour for both quantities.

In Fig. S6, panels *e, g*, we show the average degree $\langle k(N)\rangle$ and the average street length $\langle l(N)\rangle$ as a function of the number of street intersections N for the urban street network in California. In this case, while $\langle k(N)\rangle$ is compatible with a constant function of N, $\langle l(N)\rangle$ displays a slightly super-linear behaviour, which then results in slightly super-linear behaviour observed for $L(N)$ in the main text. In panels *f, h*, we show the same quantities as measured for the rural California street network, and in this case we can also observe a more inhomogeneous behaviour for both quantities.

In Fig. S7, we show the measure of the total street length $L(N)$, as measured in the historical dataset. In the left panel, we show the measure produced with the actual administrative boundary definition of Greater London, and in the right panel the same measure but over the urban street network as defined by the Jenks algorithm exposed in [3]. As we can see, the behaviour of $L(N)$, as measured for the administrative boundaries is consistently sub-linear, while it becomes linear when we consider *natural* boundaries. There is no doubt about the fact that the sub-linear behaviour emerges when we mix an urban street network with a rural street network. This figure shows the misleading results that can be obtained when we measure the properties of street networks without a proper definition for city boundaries.

For the sake of completeness, in Fig. S8, we show the statistical distribution for the logistic parameters r, τ_0 and C for the case of the UK. We show the carrying capacity analysis C, in terms of a rank statistics as it represents the city size in our approach. We report not significant statistical correlations between the different parameters.

[1] http://www.ordnancesurvey.co.uk/. Last visited 04/09/2013.
[2] http://download.geofabrik.de/north-america/us/california.html. Last visited 26/06/2014.
[3] A. Masucci, K. Stanilov, and M. Batty, London's street network dynamics since the 18th century, PLoS ONE, vol. 8 (2013), p. e69469.
[4] A. P. Masucci, K. Stanilov, and M. Batty, Exploring the evolution of London's street network in the information space: A dual approach, Phys. Rev. E, vol. 89 (2014), p. 012805.
[5] H. Makse, S. Havlin, and H. Stanley, Modelling urban growth patterns, Nature, vol. 377 (1995), pp. 608-612.
[6] H. D. Rozenfeld, D. Rybski, J. S. Andrade, Jr., M. Batty, H. E. Stanley, and H. A. Makse, Laws of population-growth, Proc. Natl. Acad. Sci. USA, vol. 105 (2008), pp. 18702-18707.
[7] H. D. Rozenfeld, D. Rybski, X. Gabaix, and H. A. Makse, The area and population of cities: New insights from a different perspective on cities, Amer. Econ. Rev., vol. 101 (2011), pp. 2205-2225.
[8] E. Arcaute, E. Hatna, P. Ferguson, H. Youn, A. Jo-

hansson, and M. Batty, Constructing cities, deconstructing scaling laws, J. R. Soc. Interface, vol. 12 (2015), p. 20140745.

[9] T. Jia and B. Jiang, Measuring urban sprawl based on massive street nodes and the novel concept of natural cities, Preprint (2011), arxiv.org/abs/1010.0541.

[10] D. Stauffer and A. Aharony, Introduction To Percolation Theory, Taylor and Francis Ltd., London, 1985.

[11] E. Arcaute, C. Molinero, E. Hatna, R. Murcio, C. Vargas-Ruiz, P. Masucci, J. Wang, and M. Batty, Hierarchical organisation of Britain through percolation theory, arXiv preprint (2015), arXiv:1504.08318.

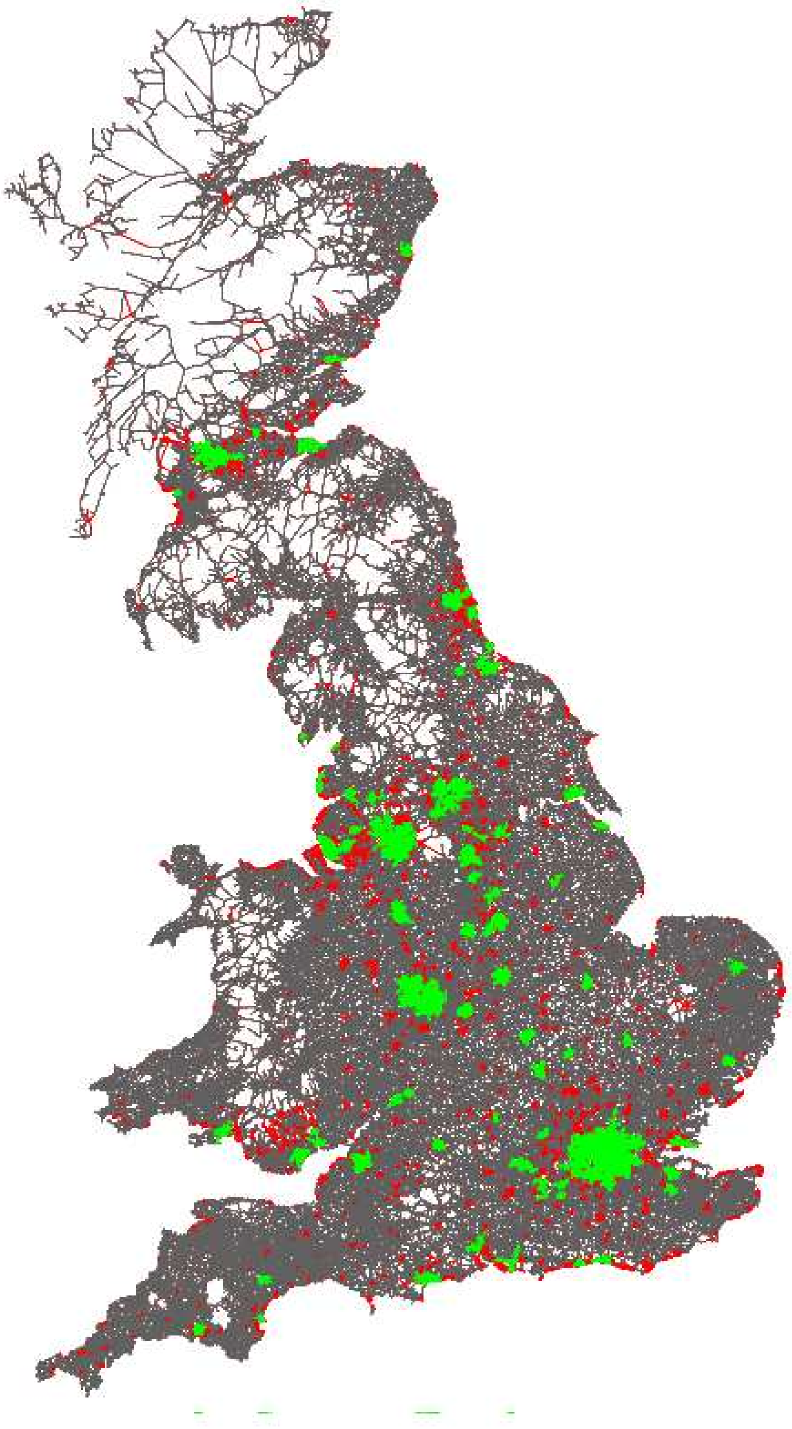

FIG. S1: The UK street network: the rural street network (grey), the urban street network (red and green), the 61 largest cities (green) employed in the analysis.

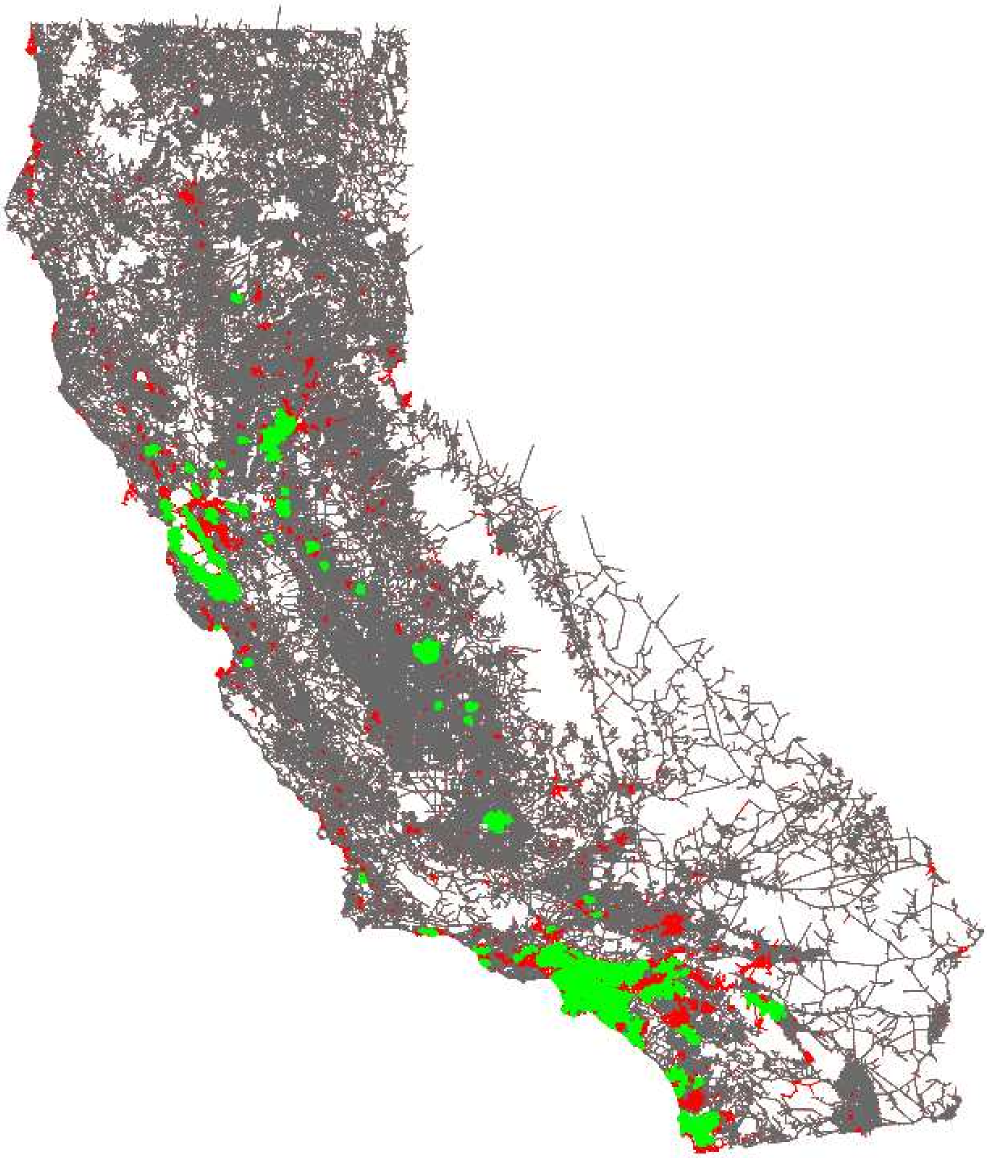

FIG. S2: The California street network: the rural street network (grey), the urban street network (red and green), the 52 cities in California which define the California urban street network (green) employed in the analysis.

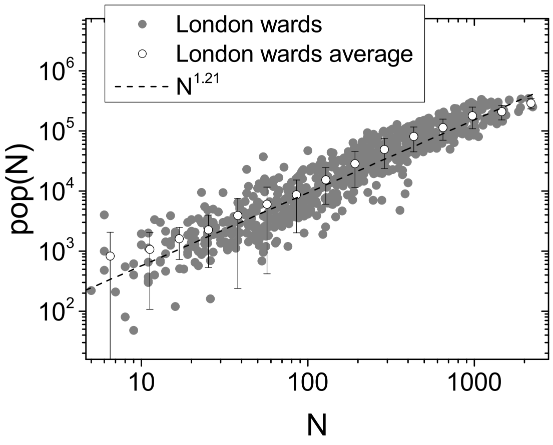

FIG. S3: Correlations between the population $pop(N)$ and the number of street intersections N for the wards of London from 1786 to 2010.

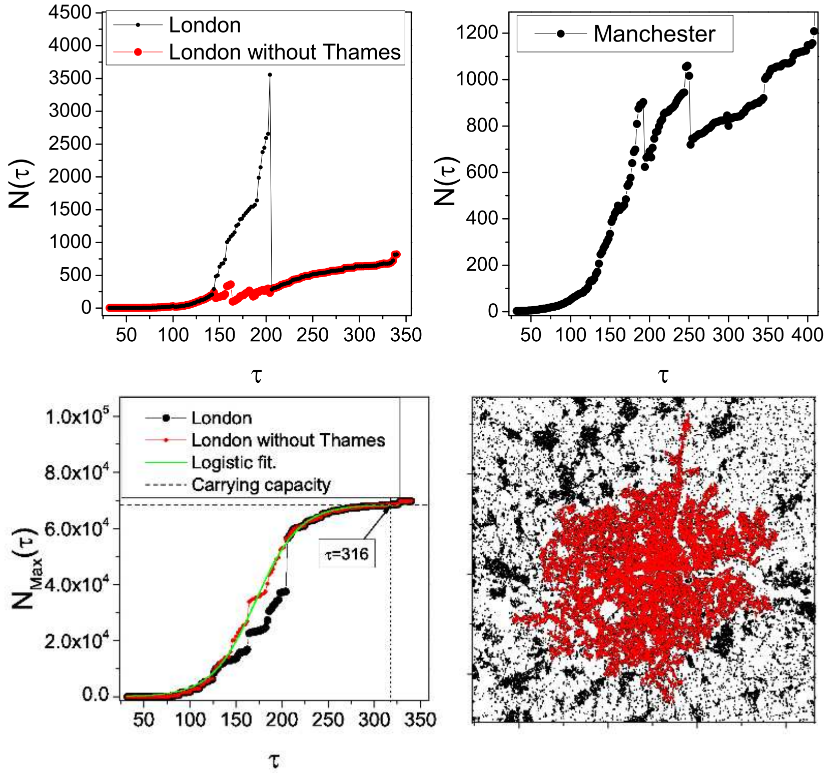

FIG. S4: Top panels: the average cluster size $N(\tau)$ of the cluster which a randomly picked intersection belongs to as a function of the threshold τ, for London (left panel) and Manchester (right panel). Bottom left panel: maximum cluster size $N_{Max}(\tau)$ as a function of the threshold τ for London. Bottom right panel: the London maximum cluster at the city condensation threshold.

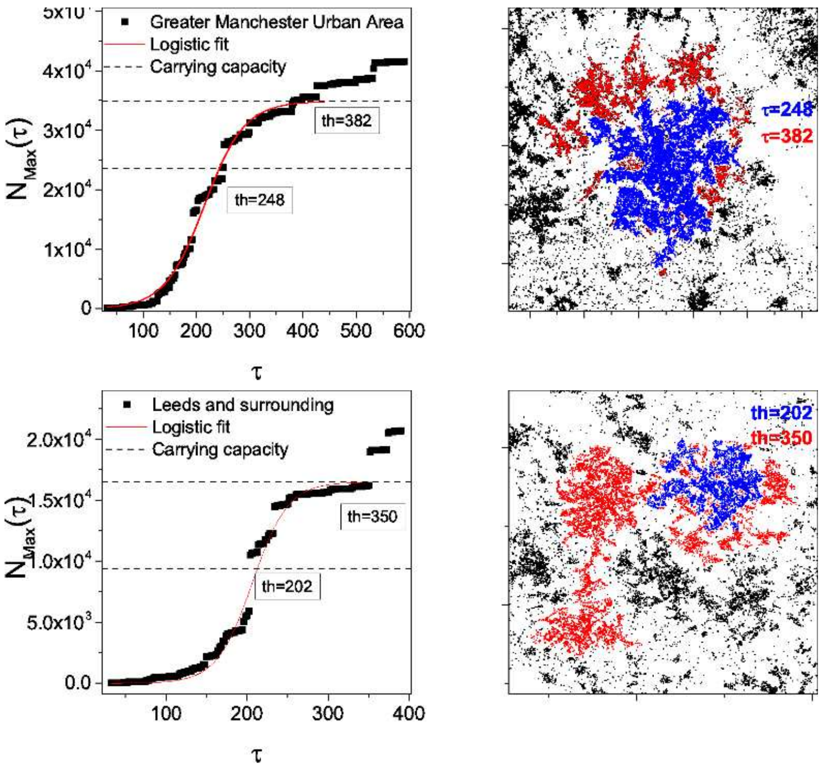

FIG. S5: Top-left panel: The maximum cluster size as a function of τ for Manchester. Top-right panel: the maximum cluster for Manchester and surrounding area for different values of τ. Bottom-left panel: The maximum cluster size as a function of τ for Leeds. Bottom-right panel: the maximum cluster for Leeds and surrounding area for different values of τ.

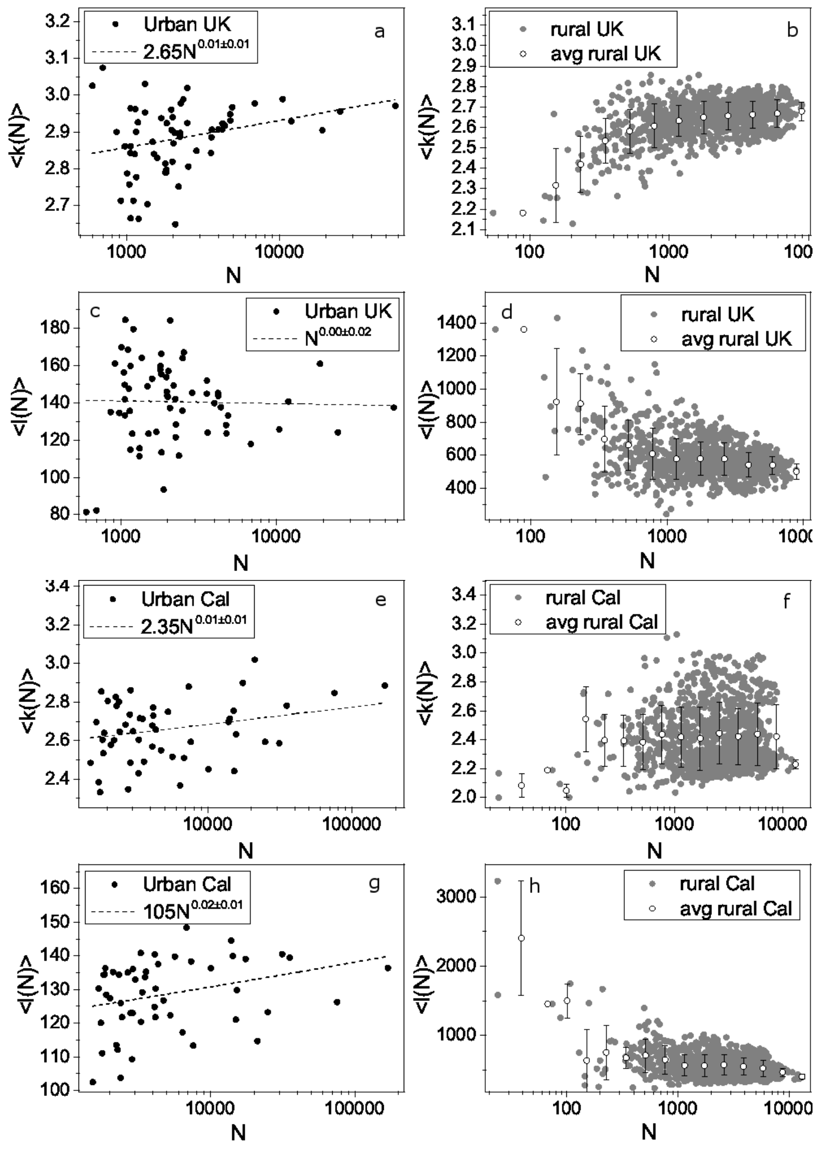

FIG. S6: *Panels a-d* The UK street network: panel a: average degree $\langle k(N) \rangle$ for the urban UK; panel b: average degree $\langle k(N) \rangle$ for the rural UK; panel c: average street length $\langle l(N) \rangle$ for the urban UK; panel d: average street length $\langle l(N) \rangle$ for the rural UK. *Panels e-h* The California street network: panel e: average degree $\langle k(N) \rangle$ for urban California; panel f: average degree $\langle k(N) \rangle$ for rural California; panel g: average street length $\langle l(N) \rangle$ for urban California; panel h: average street length $\langle l(N) \rangle$ for rural California.

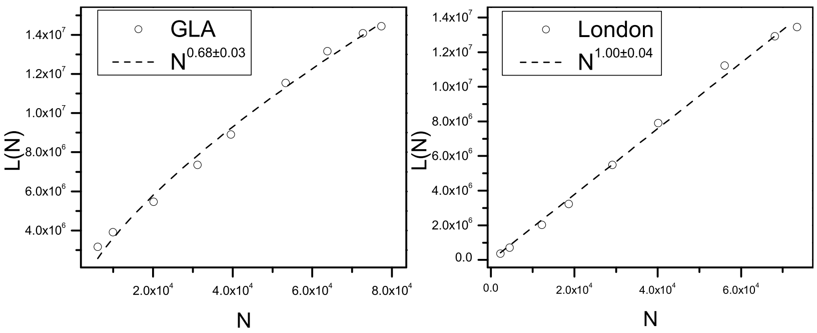

FIG. S7: Figure taken from [3]: in the left panel the total street length $L(N)$ for the historical dataset as measured in the Greater London administrative boundary. In the right panel $L(N)$ as measured in the same dataset, when the city is extracted using a *natural* boundaries procedure.

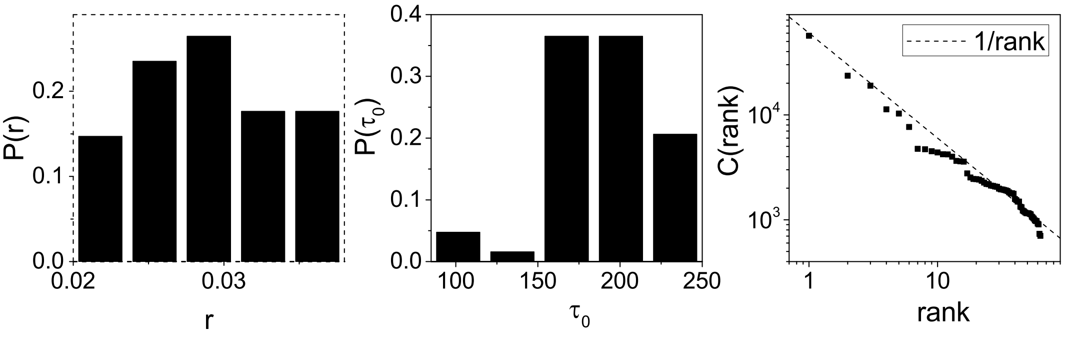

FIG. S8: Analysis for the logistic parameters for the UK. In the left panel the frequency distribution $P(r)$ for the growth parameter r. In the central panel the frequency distribution $P(\tau_0)$ for the inflection point τ_0. In the right panel the frequency rank statistics $C(rank)$ for carrying capacity C.
